# Incidence of atrial fibrillation in different major cancer subtypes: a Nationwide population-based 12 year follow up study

**DOI:** 10.1186/s12885-019-6314-9

**Published:** 2019-11-14

**Authors:** Christina Boegh Jakobsen, Morten Lamberts, Nicholas Carlson, Morten Lock-Hansen, Christian Torp-Pedersen, Gunnar H. Gislason, Morten Schou

**Affiliations:** 1 Department of Cardiology, Copenhagen University Hospital Gentofte-Herlev, Kildegaardsvej 28, 2900 Hellerup, Denmark; 2 Department of Nephrology, Copenhagen University Hospital Gentofte-Herlev, 2730 Herlev, Denmark; 3 Department of Health, Science and Technology, Aalborg University, Aalborg, Denmark

**Keywords:** Atrial fibrillation, Arrhythmia, Cancer, Malignancy

## Abstract

**Background:**

The prevalence of both atrial fibrillation (AF) and malignancies are increasing in the elderly, but incidences of new onset AF in different cancer subtypes are not well described.The objectives of this study were therefore to determine the incidence of AF in different cancer subtypes and to examine the association of cancer and future AF.

**Methods:**

Using national databases, the Danish general population was followed from 2000 until 2012. Every individual aged > 18 years and with no history of cancer or AF prior to study start was included. Incidence rates of new onset AF were identified and incidence rate ratios (IRRs) of AF in cancer patients were calculated in an adjusted Poisson regression model.

**Results:**

A total of 4,324,545 individuals were included in the study. Cancer was diagnosed in 316,040 patients. The median age of the cancer population was 67.0 year and 51.5% were females. Incidences of AF were increased in all subtypes of cancer. For overall cancer, the incidence was 17.4 per 1000 person years (PY) vs 3.7 per 1000 PY in the general population and the difference increased with age. The covariate adjusted IRR for AF in overall cancer was 1.46 (95% confidence interval (CI) 1.44–1.48). The strength of the association declined with time from cancer diagnosis (IRR_0-90days_ = 3.41 (3.29–3.54), (IRR-_180 days-1 year_ = 1.57 (CI 1.50–1.64) and (IRR_2–5 years_ = 1.12 (CI 1.09–1.15).

**Conclusions:**

In this nationwide cohort study we observed that all major cancer subtypes were associated with an increased incidence of AF. Further, cancer and AF might be independently associated.

## Background

Atrial fibrillation (AF), repeatedly named the new epidemic in cardiology [1, 2], affects 1.5–2% of the general population and prevalence is likely to double within the next 50 years [3]. AF is a major risk factor for developing cardiovascular complications, and is associated with a 5-fold increased risk of stroke, a 3-fold incidence of heart failure and an increased mortality [3]. Besides age, several cardiovascular conditions such as hypertension, valvular heart disease and heart failure, as well as non-cardiovascular conditions such as diabetes, chronic pulmonary disease, obesity, surgery and alcohol are established risk factors for AF [3–6]. Cancer is associated with an increased inflammatory activity [7–9] and paraneoplastic manifestations [10, 11]. However, it is unknown whether cancer is an independent risk factor for development of AF [12].

Knowledge regarding the association of cancer and AF is very sparse and it has only been examined in a few studies. One case-control study [13] observed an increased risk of AF in all cancer subtypes when compared to non-cancer patients, but only within the first 90 days after the cancer diagnosis. Another observational cohort study among women found similar results, with increased risk of AF the first 3 months following a cancer diagnosis [14]. Finally a small cohort study [15] has described an increased association between breast cancer or colorectal cancer and future AF. Whether the increased risk of AF is associated with all subtypes of cancer or only limited to certain subtypes is unknown at present. Furthermore, the incidence, clinical significance and appearance of new onset of AF in relation to the time of the cancer diagnosis are also unknown [12].

The objectives of this study are, therefore, to describe the incidence of AF and its appearance in relation to time from diagnosis of cancer in different cancer subtypes, and to test whether cancer is an independent risk factor for AF or whether it is explained by comorbidity associated with cancer.

## Methods

### Registries

In Denmark every citizen has a unique personal registration number. We used this number to link individual data across several nationwide databases: The National Patient Registry classifies all hospital contacts with regards to the International Classification of disease (ICD). Procedures performed are coded according to the Nordic Medical Statistics committee of Surgical Procedures. The National Prescriptions registry provides information regarding the dose, number of tablets and the date of dispensing according to the Anatomical Therapeutic Chemical Classification system. Vital status, gender, and cause of death according to the ICD 10th revision were acquired from the Danish Personal Registration System and the National Causes of Death Register. Diagnosis, pharmacotherapy, surgical procedures and comorbidities used to identify and define the population are available in Additional file 4.

### Study population

**Fig. 1:**
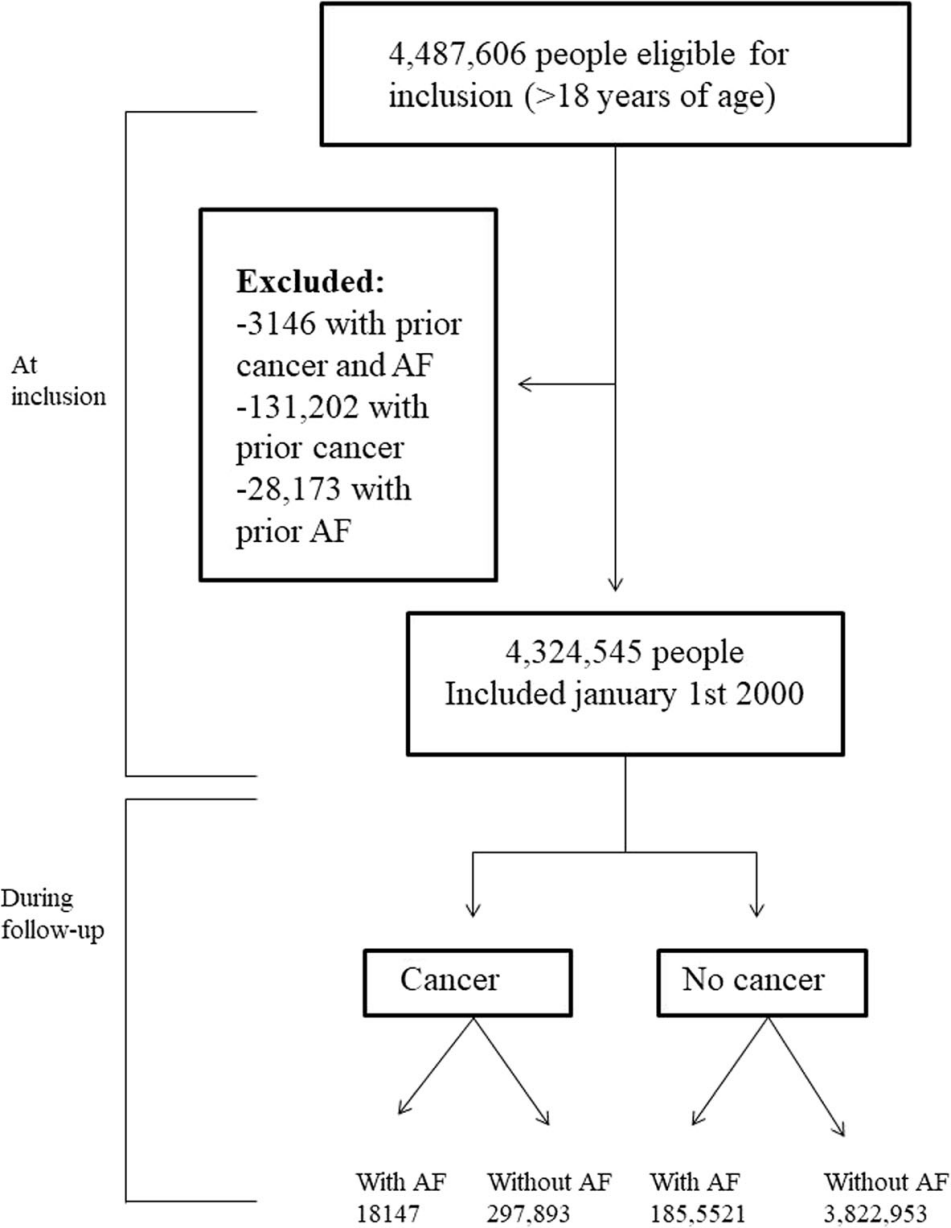
The study population. Flowchart of the study population

All Danish citizens aged 18 years of age or above were included January 1, 2000. Individuals diagnosed with AF or cancer prior to inclusion were excluded, hence at inclusion all individuals were categorized as the general population (i.e. not having cancer). If diagnosed with cancer during follow-up, subjects changed status from general population to the cancer group at the date of their cancer diagnosis. Thus in the statistical analyses, patients who developed cancer during the study, did not appear as a part of the endpoint results in the general population. The cohort was followed until either the debut of AF, emigration, death, or December 31st 2012, whichever came first. Figure 1 depicts the study population. Patients diagnosed with cancer were sub grouped according to type of cancer. For patients registered with more than one type of cancer, only the first cancer diagnosis was included in the study.

### Comorbidity and pharmacotherapy

The prevalence of the following comorbidities were characterized at inclusion: Ischemic stroke, myocardial infarction, previous embolus, liver disease, abuse of alcohol or psychoactive substances, previous bleeding, vascular disease, chronic renal failure, chronic obstructive pulmonary disease, thyroid disease, heart failure, and hypertension. Heart failure was defined as a prior diagnosis of heart failure plus the use of loop diuretic as done previously [16, 17]. Hypertension was defined as a combination treatment with a least two antihypertensive drugs as done previously [16, 18, 19] .

Pharmacotherapy was characterized for the following drugs; loop diuretics, renin-angiotensin system inhibitors, beta-blockers, aldosterone antagonists, thiazides, statins, anti-platelet therapies, vitamin K antagonists, anti-diabetes medication, and inhalation therapies for obstructive pulmonary diseases.

We report comorbidities and pharmacotherapy at the time of inclusion, and also at the time of the cancer diagnosis. Prescriptions redeemed within 180 days prior to inclusion and cancer diagnosis defined treatment.

### Study outcomes

AF was identified by the ICD-10 diagnosis code ‘DI48’ from The National Patients Registry. The diagnostic coding for AF has previously been validated; where the positive predictive value was 93%, and results were comparable between AF primary and or secondary diagnoses [20]. In our main analyses, any primary or secondary diagnosis of AF was included in order to capture all hospitalizations related to AF. However, this captures random findings of non-symptomatic AF in relation to acute conditions being the primary reason for hospitalization. To ensure that our results were not biased, we furthermore conducted a sensitivity analysis, where we solely included cases of AF registered as the primary diagnosis.

### Statistical analysis

All presented rates are crude incidence rates (IR) calculated as events per 1000 person-years (PY) with 95% confidence intervals (CIs). Additionally, the incidence rates were stratified by sex and presented with continuously updated patient age (and age group accordingly). The lexis-macro (http://www.bendixcarstensen.com/Lexis/Lexis.sas last accessed January 21, 2016) was used for all analyses and included three time scales; calendar time (bands were split in 1 year periods after 1th January 2000), age (bands were split in 1 year periods according to date of birth) and time from cancer diagnosis (bands were split after 3, 6, 12, 24, 60 and > 60 months respectively from date of cancer diagnosis).

Multivariable Poisson regression models were fitted to estimate incidence rate ratios (IRRs) of AF in cancer patients with the general population as reference. We defined two models. (I) An analysis only adjusted for age and gender, and (II) a fully adjusted time dependent analysis (i.e. continuously assessment and update of characteristics during the entire study period)) adjusted for age, gender, calendar time, and including adjustment for the above mentioned comorbidities (ischemic stroke, myocardial infarction, previous embolus, liver disease, abuse of alcohol or psychoactive substances, previous bleeding, vascular disease, chronic renal failure, chronic obstructive pulmonary disease, thyroid disease, heart failure, and hypertension) and pharmacotherapy (loop diuretics, renin-angiotensin system inhibitors, beta-blockers, aldosterone antagonists, thiazides, statins, anti-platelet therapies, vitamin K antagonists, anti-diabetes medication, and inhalation therapies for obstructive pulmonary diseases). As AF is known to be seen postoperatively, [6], the regression model were further adjusted for all major gastric-, orthopedic-, thoracic and cardiac surgeries, including cancer-related surgeries, performed within 30 days prior to an AF diagnosis. Only surgeries requiring hospitalization for 3 days or more were included, (assuming severe disease or surgery more prone to AF development). The adjustments for major surgeries were used for both the cancer population and the non-cancer population. In the fully adjusted model, each patient following inclusion was split into multiple observations according to the criteria above. i.e. three time scales, date of dispensed new prescription or comorbidity and surgery. All patients were followed from inclusion until a diagnosis of AF, death, emigration and study end, whichever came first.

To ensure a potential association between AF and cancer was not solely influenced by AF being diagnosed at time of a cancer diagnosis, we defined a third model, where we performed analyses within time periods from cancer diagnosis i.e. “0–90 days”, “90–180 days”, “180–365 days”, “1–2 years”, “2–5 years”, and “> 5 years”.

A two tailed *P*-value ≤0.05 was considered significant. We tested for relevant interaction and no clinical relevant violation of model assumptions were found (linearity, goodness-of-fit). Data management and statistical analyses were performed using SAS version 9.4.

### Ethics

The study has been approved by the Danish Data Protection Agency (2007-58-0015 / local ref. no. GEH-2014-013, I-Suite no: 02731. Data was made available to us, so no individuals could be identified. As a retrospective registry-based study, Danish law does not require ethical approval.

## Results

### Study population

A total of 4,324,545 people from the general Danish population were included on January 1st, 2000. During 12 years of follow-up, 316,040 persons (7.3%) were diagnosed with cancer with a female predominance of 51.5% and a median age at disease onset of 67.0 years (IQR 58.0–75.8). Figure 1 shows a flow chart of the study population and Table 1 shows clinical characteristics of the patients who developed cancer and of the general population.

**Table 1:** Baseline characteristics

Clinical characteristics	General population (*n* = 4,324,545)	Cancer population (*n* = 316,040)
Male sex	2,176,883 (50.3)	153,258 (48.5)
Median (SD*) age (years)	44.8 (18.0)	67.0 (13.3)
Comorbidity:		
Stroke	63,619 (1.5)	19,589 (6.2)
Ischemic heart disease	64,596 (1.5)	17,297 (5.5)
Heart failure	22,271 (0.5)	6226 (2.0)
Hypertension	179,184 (4.1)	111,157 (35.2)
Vascular disease	83,119 (1.9)	25,590 (8.1)
Previous bleeding	27,104 (0.6)	36,996 (11.7)
Chronic obstructive pulmonary disease	19,639 (0.5)	19,127 (6.1)
Chronic kidney disease	36,595 (0.9)	8962 (2.8)
Misuse of alcohol or psychoactive substance	73,056 (1.7)	17,533 (5.6)
Hyperthyroid disease	29,945 (0.7)	9704 (3.1)
Previous embolus	81,350 (1.9)	26,708 (8.5)
Liver disease	27,104 (0.6)	9395 (3.0)
Pharmacotherapy		
Calcium channel blocker	154,619 (3.6)	43,591 (13.8)
ACE† inhibitors	173,182 (4.0)	67,737 (21.4)
Beta blockers	158,308 (3.7)	42,629 (13.5)
Spironolactone	18,023 (0.4)	9132 (2.9)
Loop diuretic	118,914 (2.8)	35,159 (11.1)
Thiazide diuretic	168,222 (3.9)	43,289 (13.7)
Aspirin	175,178 (4.1)	60,598 (19.2)
Clopidogrel	1444 (0.03)	4488 (1.4)
Warfarin	1558 (0.5)	7376 (2.3)
Digoxin	41,430 (1.0)	6852 (2.2)
Cholesterol-lowering drug	62,599 (1. 5)	50,301 (15.9)
Glucose-lowering medication	8791 (2.9)	22,979 (7.2)
Inhalation medication	222,169 (5.1)	40,355 (12.8)

Baseline characteristics for the general population and the cancer population. Values are numbers (percentages) unless stated otherwise

*SD = standard deviation, †ACE = angiotensin converting enzyme

### Incidence of AF after a cancer diagnosis: sex and cancer type stratified analyses

**Fig. 2:**
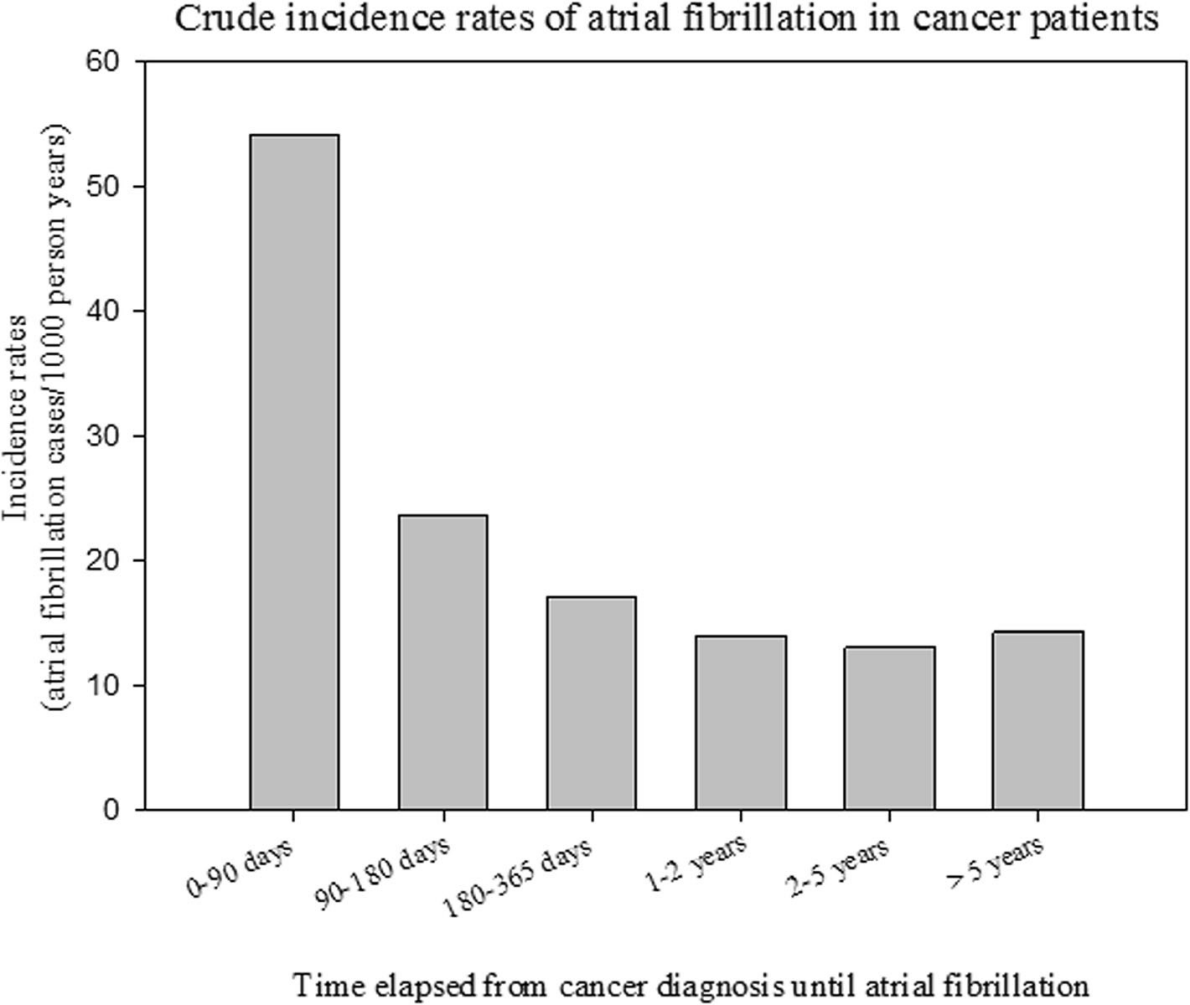
Crude incidence rates of atrial fibrillation in cancer patients. The rates are stratified according to time from cancer diagnosis until AF diagnosis

**Fig. 3:**
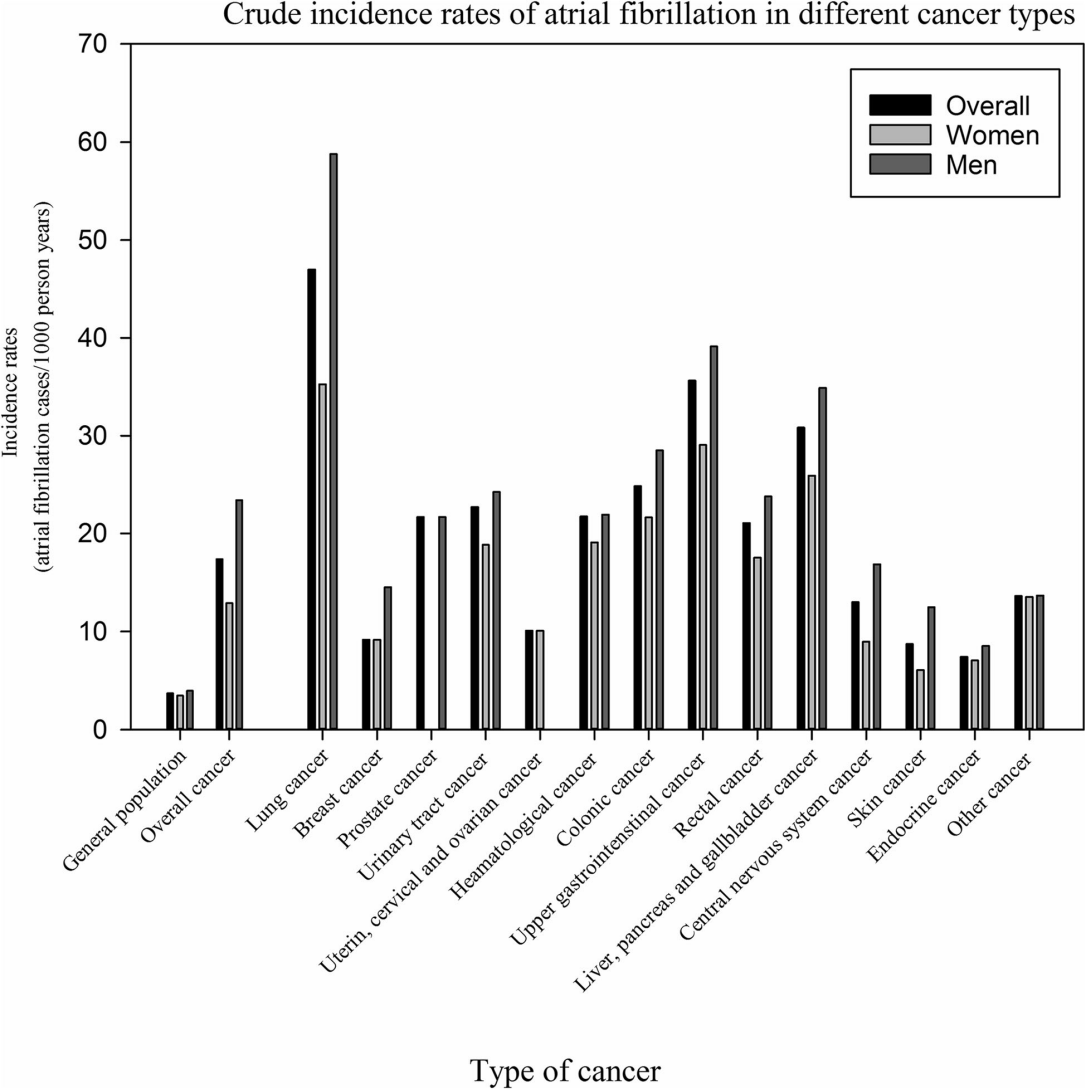
Crude incidence rates of atrial fibrillation in the general population, in overall cancer patients and in individual types of cancer. The rates are shown for the whole population and for women and men independently

**Fig. 4:**
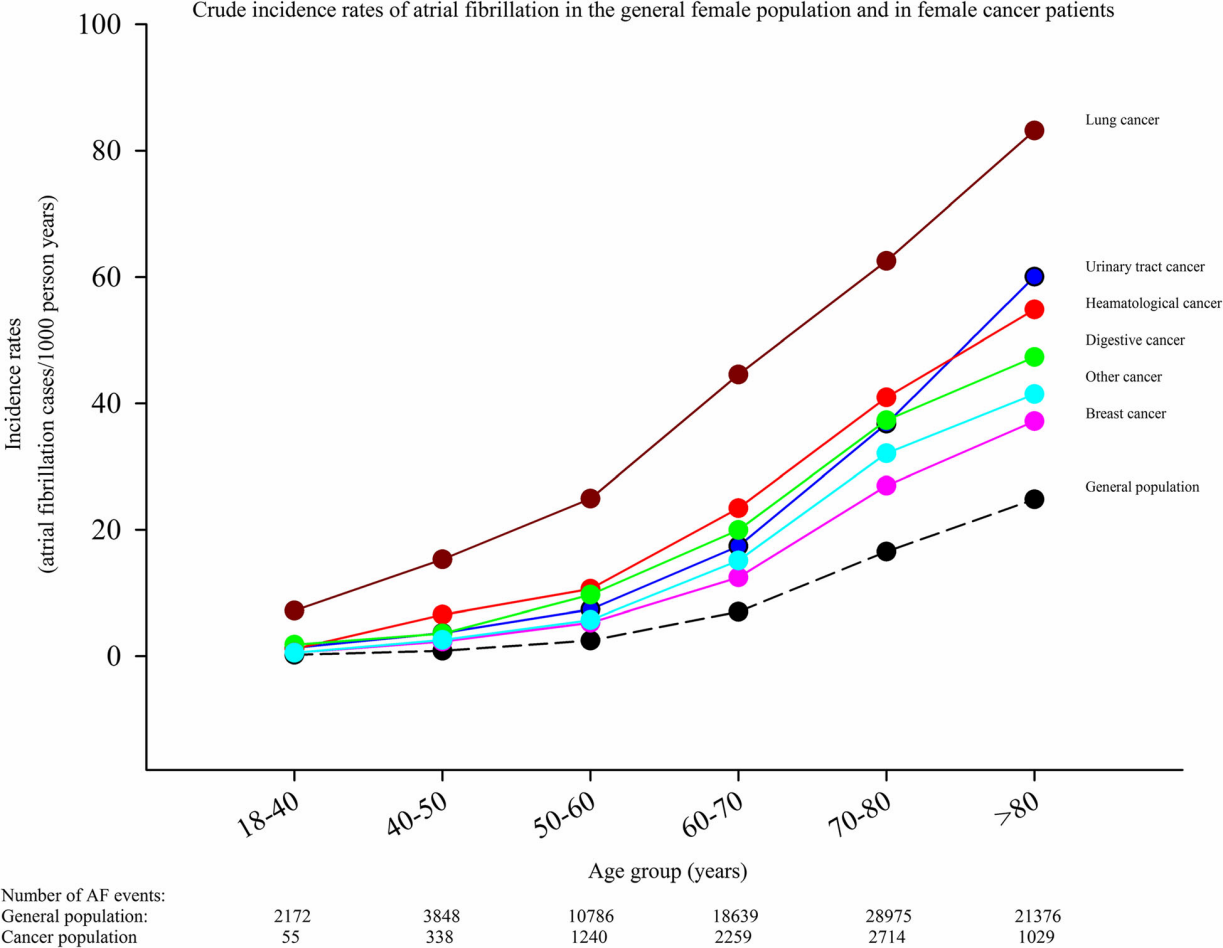
Crude incidence rates of atrial fibrillation in females. Crude incidence rates of atrial fibrillation in the general female population and in female patients with cancer. The model is stratified according to age and follow-up time

**Fig. 5:**
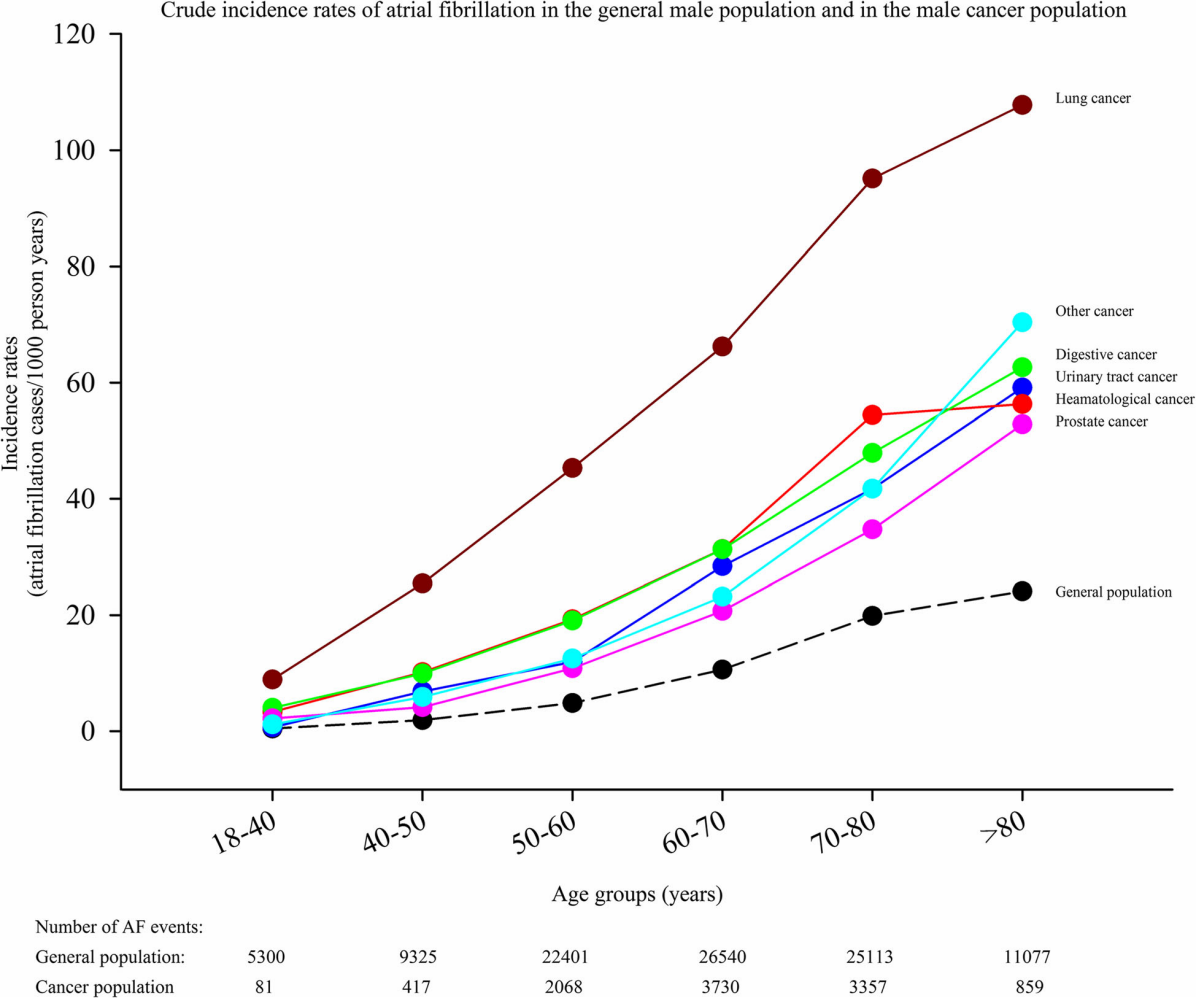
Crude incidence rates of atrial fibrillation in males. Crude incidence rates of atrial fibrillation in the general male population and in male patients with cancer. The model is stratified according to age and follow-up time

The crude incidence rates of AF in cancer patients stratified according to time from cancer diagnosis until AF diagnosis are shown in Fig. 2. Figure 3 shows the crude incidence rates according to cancer and sex. The incidence rate is highest for AF diagnosed within 90 days from the date of the cancer diagnosis, but remains higher than the incidence rate for the general population without cancer for more than 5 years. For every cancer type, the incidence rates of AF is greater compared with the incidence rate of the general population. In the general population the incidence of AF was 3.7 per 1000 person years (PY) compared to 17.4 per 1000 PY in patients with a cancer diagnosis (excluding the first 90 days the rate was 13.7 per 1000 PY). The highest incidence was observed in lung cancer in both men (58.7 per 1000 PY) and women (35.3 per 1000 PY). Figures 4 and 5 show the gender-specific crude incidence rates of AF in different major cancer types compared to the general population. Both figures illustrate that the incidence of AF in all subtypes of cancer increases as function of age and follow up time for both women and men.

### Association between cancer and incidence AF: Poisson regression analyses

**Fig. 6:**
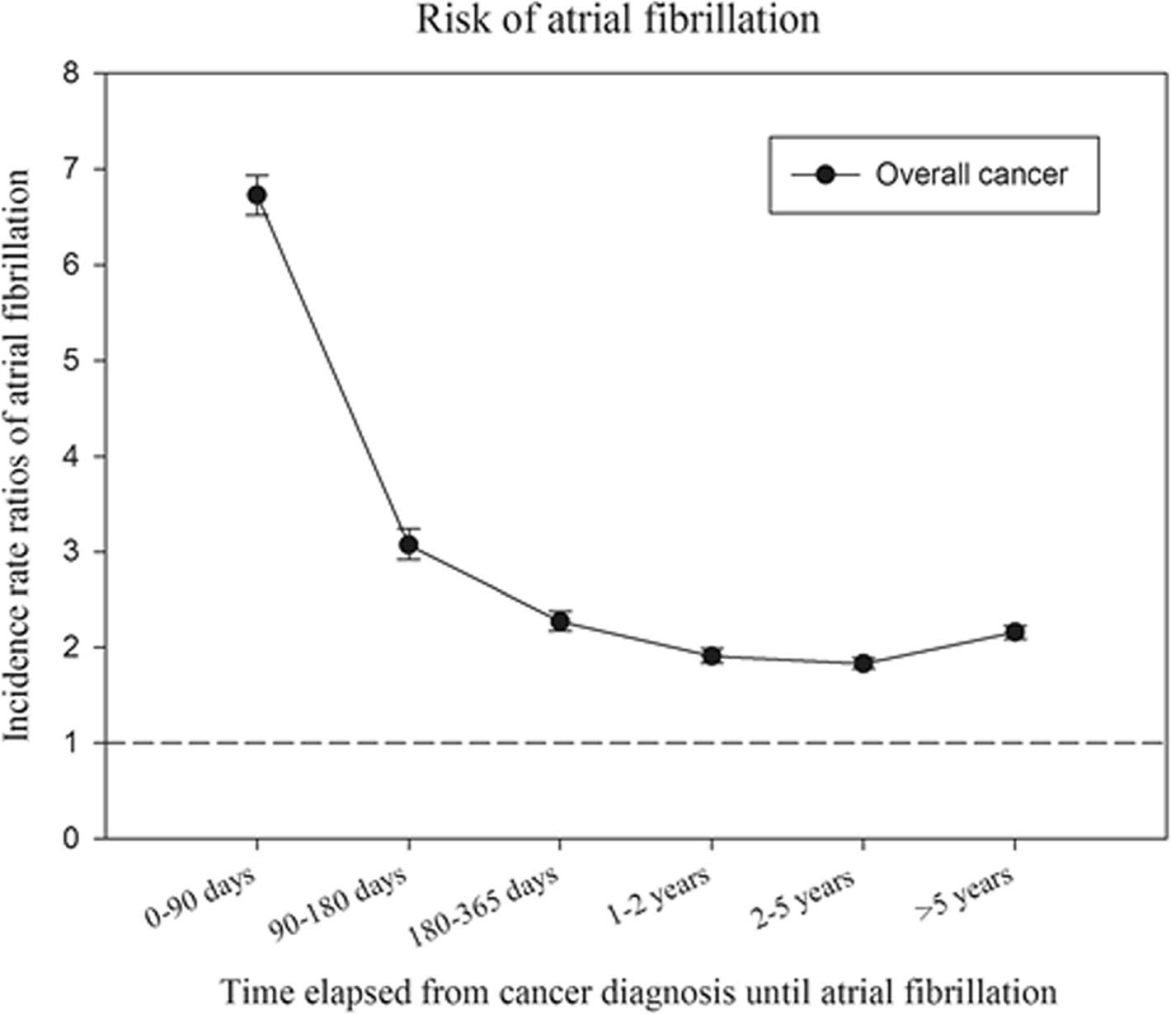
Incidence rate ratios of atrial fibrillation. Risk of atrial fibrillation among patients with cancer compared to persons without cancer - stratified according to time from cancer diagnosis until AF diagnosis. The model is adjusted for sex and age

**Fig. 7:**
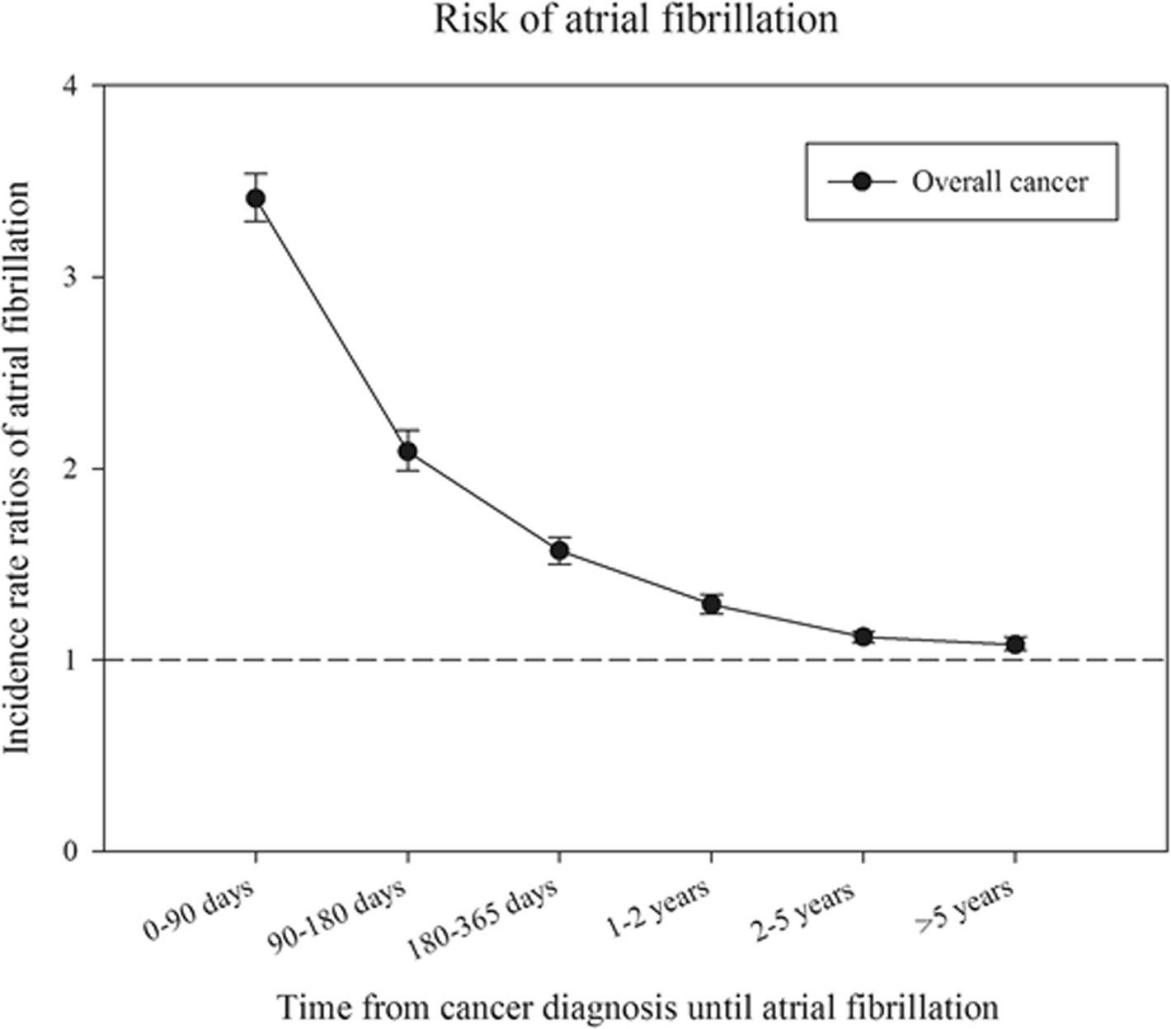
Incidence rate ratios of atrial fibrillation. Risk of atrial fibrillation among patients with cancer compared to person without cancer stratified according to time from cancer diagnosis until AF diagnosis. The model is adjusted for time, age, sex, comorbidities and earlier surgeries

Age- and sex- adjusted and fully-adjusted (adding calendar year, sex, age, former surgeries, comorbidities and pharmacotherapy) incidence rate ratios (IRR) of AF are shown for overall cancer in Figs. 6 and 7, respectively. The figures illustrate that the association between overall cancer and AF is highest within the first 90 days, but it remains significant over time.

IRRs over time according to specific cancers largely resembled main analysis. (Additional file 1: Table S1) Notably, the IRRs for lung cancer and hematological cancer are markedly increased within the first 90 days and remain significantly increased 5 years after cancer diagnosis.

### Additional analyses: association between cancer and future AF as primary diagnosis

We also conducted a sensitivity analysis, where only AF as a primary diagnosis was used as an outcome, to ensure that our results were not biased by hospitalization for other reasons where AF was found by chance. In these additional analyses, we found the IRR for all cancer forms to be 1.23 (1.20–1.26) – compared to 1.46 (1.44–1.48) when AF as a secondary diagnosis was included. Contrary to the main analyses, the sub-analysis only found a significantly increased IRR within the first 5 years after the cancer diagnosis and thus not an increased risk more than 5 years after (Additional file 2: Table S2). For some cancers (i.e liver, pancreas or gallbladder, rectal, skin and urinary tract cancers), the associated risk of AF was comparable to the background population 2 years following a diagnosis of cancer.

## Discussion

Main findings of the present study are that in both men and women, for all ages and major subtypes of cancer, a cancer diagnosis is associated with an increased incidence of new-onset AF. Second, cancer and future AF seems to be independently associated. Finally and importantly, AF appears more frequent in cancer patients up to 5 years following cancer diagnosis.

### Other studies and mechanism(s)

Knowledge upon this topic is sparse. No previous studies have investigated the incidence of AF in an unselected cohort of patients with different forms of cancer. The association between cancer and AF has only previously been examined within subgroups of cancer or in connection with surgeries in smaller clinical studies. Thus there are many open issues concerning the burden of AF in cancer patients [12]. Our findings do, therefore, add significant clinical data to the knowledge on the relationship between cancer and new-onset AF. The mechanism(s) underlying the association between cancer and AF cannot be deduced based on our results and may differ between the different cancer forms, e.g. the strong correlation between lung cancer and AF suggests an influence of direct tumor growth. It has also been shown, that inflammatory markers such as C-reactive protein are elevated in AF. As inflammation also plays a large role in cancer, it is possible that cancer could lead to AF through a systemic inflammatory state [7–9]. Finally the presence of paraneoplastic syndromes and neurohormonal activity could also lead to AF [10, 11]. The statistical association is, therefore, biological plausible and more research in the mechanism(s) are needed.

### Incidence of AF

The incidence of AF stratified according to time and subtypes of cancer is shown in Figs. 2 and 3. It may be speculated that the observed difference is explained by age, since cancer patients were older than the general population (Table 1). We, therefore, performed age and sex stratified analyses which confirmed that incidence of AF was greatest in the cancer population (Figs. 4 and 5). We observed a smaller difference in the incidence of AF between cancer patients and the general population than previously observed [15]. This may be explained by differences in design, since we excluded known AF which was not the case in the aforementioned study. Despite the inherent limitations in our study, incidence rates (both within the first 90 days and beyond) are markedly increased and should raise concern from physicians treating patients with malignancies. Rates of AF in patients with cancer are equivalent to rates found in patients with diabetes and rheumatoid arthritis [16, 21].

When looking at our entire study population, cancer patients as well as non-cancer patients, we observed a little lower incidence of AF than observed in other AF studies [22, 23]. However, this can be explained by the several factors; first of all by the differences in how AF is defined. We defined AF by ICD10 codes, while the Framingham Heart Study [22] had access to e.g. Holter monitoring and electrocardiograms for all of their participants. Additionally the participants of the Framingham Heart Study were between 50 and 89 years of age and thus comparably older. As such, the observed incidence of AF also reflects the correlation of age with risk of AF. We observed the same incidence of AF in elderly (> 80 years) general population as in the Rotterdam Study (23).

### The association between cancer and AF

Our findings support the current evidence [12] that an association between cancer and future AF exists. The correlation has up until now primarily been investigated with regards to colorectal and breast cancer, but our results demonstrates that 12 out the 13 examined cancer forms (including pulmonary cancer, prostate cancer, urinary tract cancer and hematological cancer) were associated with increased risk of developing AF (Additional file 3: Table S3). Additionally, the non-significant association between endocrine cancer and future AF could be due to under powering; hence incidence of endocrine cancer was rare in our population (data not shown).

Notably, two prior studies [13, 14] demonstrated that the greater risk of AF in cancer patients was limited to the initial 90 following cancer diagnosis; thus, indicating that the association could be due to observations bias. This bias would emerge as asymptomatic cancer patients have a greater chance of being diagnosed with AF than asymptomatic otherwise healthy individuals. To minimize the hazard of such bias we studied time from date of cancer diagnosis until time of potential AF. Our results show the same tendency; association between cancer and AF is strongest within the first 90 days following the cancer diagnosis, thus also indicating the presence of some degree of observation bias. However, the association within our study remains significant as long as 5 years following the cancer diagnosis. Therefore, the presence of surveillance bias is unlikely to be the solely explanation of our findings. The reason that we find a significant association beyond 90 days could be due to larger sample size (316,040 cancer patients).The prognosis of cancer improves considerably these years [24] and with a considerable burden of AF in the elderly showed in our study among others, awareness of the development of AF is important even in cancer patients surviving a 5 year milestone.

As seen in Additional file 2: Table S2 and Additional file 3: Table S3 the strongest association between subtype of cancer and AF goes for lung cancer despite a poor prognosis in these patients. On the other hand, prostate cancer has the lowest significantly association to AF despite a relatively good prognosis. It may, therefore, be argued that severity of the cancer disease or anatomical location of the tumor is important factors for development of AF. Also, time from the diagnosis of cancer is an important factor to consider, especially concerning cancer in the abdominal region.

Another possible explanation is the effect of specific treatment for specific cancers, first line therapies for prostate cancer includes local radiation, hormonal therapy and less invasive surgery (prostatectomy), which all should be relatively less linked with development of AF.

It is plausible, that the reason for the observed increase in incidence of AF the first 90 days following cancer diagnosis could be related to the subclinical progression of cancer prior to diagnosis. Patients presenting with newly diagnosed cancer are predominantly subject to the accumulated effects of prolonged disease activity. As such, the debut of AF shortly after cancer diagnosis could reflect the result of such prolonged exposure.

### Radiation and chemotherapy

Whether AF could emerge as a side effect to other sorts of treatments of the cancer disease, such as radiation therapy or chemotherapy have not been investigated in this study. Especially with regards to lung cancer and breast cancer it is possible that radiation therapy could be involved in causing AF due to direct radiation against the heart. However, looking at the individual IRR of the cancer types, the association is actually higher for cancer in the digestive system and cancer in the central nervous system than for breast cancer suggesting that the association in some cancer types must be explained by other factors than radiation therapy.

Furthermore, due to the fact that chemotherapy is considered in-hospital treatment in Denmark, our registries do unfortunately not contain exact data with regards to type of chemotherapy or the duration of treatment. Although AF incidence was especially pronounced within the first 90 days, which could be the possible effect of certain types of chemotherapies, the association of AF risk and cancer was still elevated beyond 90 days. The scope of this study was to assess the association (and not the causal path way) between different types of cancers AF on a population level. Studies on the impact of specific chemotherapies on AF risk are needed as no information on chemotherapies was available for the current study. This important limitation should be recognized when interpreting our findings.

### Strengths and limitations

The strengths of this study include the inclusion of the complete Danish population independent of age, gender, ethnicity and participation in health insurance programs. Due to these aspects the risk of information and referral bias is reduced. Still, some limitations should be considered when interpreting the results.

The largest limitation in the present study is the dependence on registry data. The identification of our study end point, AF, relied on the presence of an AF discharge code, but not by a validated electrocardiogram. However, the positive predictive value of the diagnosis of atrial fibrillation and flutter has been reported to be 93% [20] and the accuracy of other hospital registry diagnoses are similar high [25].

In general, post-operative AF is one of the most common complications to surgeries, cardiac as well as none cardiac surgeries. However, we have sought to eliminate this potential confounding by adjusting for all major surgeries The association is, therefore, not likely to be driven only by former surgeries.

Since the symptoms of AF are perceived in very individual ways, and sometimes not at all, it is very likely that some people were suffering from subclinical AF, which may have resulted in misclassification of AF. It may be speculated that cancer patients are more aware of symptoms, and subclinical AF, therefore, is more frequent in the general population, who ignore symptoms. This may have biased our results in favor of an association between cancer and AF towards one. Further, due to more regular medical examinations in patients with cancer, the risk of surveillance bias will emerge in relation to subclinical AF in the no AF group of cancer patients. When the analyses were limited to cases where AF was the primary cause to hospitalization and thus trying to eliminate AF cases randomly diagnosed in relation to cancer control, the association was less strong, possibly due to a smaller number of outcomes. However, tendency of the results were overall the same. Our results are, therefore, not solely the effect of surveillance bias and misclassification.

As seen in Table 1 the groups differ from each other with respect to clinical characteristics; however the multivariable regression analyses have been adjusted for these differences in characteristics.

Furthermore, our registries do not provide information regarding AF diagnoses solely treated by general practitioners. Patients with uncomplicated and subclinical AF who never have been hospitalized in relation to AF will therefore not be included in our study. This could potentially lead to an underestimation of the incidence of AF in both groups.

Thus, residual confounding cannot be excluded since clinical parameters such as blood pressure and HBA1C were not measured. We were unable to adjust for potential clinical confounders such as obesity and smoking and as some cancer forms are associated with a higher prevalence of smoking or obesity, availability of such information could have changed the risk estimates. We did not echocardiographic data on valve disease. Some of this unmeasured confounding was probably accounted for in the analyses by adjustment for lifestyle associated diseases. It is, therefore, unlikely that our results only originate from lack of data on other potential confounders.

## Conclusions

Among more than 300,000 individuals diagnosed with cancer, the incidence and risk of new-onset AF was significantly increased compared to the general population. Especially lung and gastrointestinal cancers were associated with considerable high rates. The association was observed for 12 out of 13 cancer types and the estimates decreased over time. Further, cancer and AF might be independently associated. Future studies need to focus on prognosis, risk of thromboembolic complications and treatment, but also on risk factors, pathogenic mechanisms and the impact of different treatment regimes for development of AF in cancer patients.

## Supplementary information

**Additional file 1: Table S1.** Incidence rate ratios of atrial fibrillation in overall cancer and in different cancer types divided into different time periods. The model is adjusted for time, age, sex, comorbidities and earlier surgeries.

**Additional file 2: Table S2.** Incidence rate ratios of atrial fibrillation in overall cancer divided into different time periods. Atrial fibrillation here only as the primary diagnosis. The model is adjusted for time, age, sex, comorbidities and earlier surgeries.

**Additional file 3: Table S3.** Incidence rate ratios of atrial fibrillation in overall cancer and in the different cancer types. The model is adjusted for time, age, sex, comorbidities and earlier surgeries.

**Additional file 4.** ICD and ATC codes for identification of cancer diagnosis, surgeries, comorbidities and pharmacotherapy.

## Data Availability

Data used in this study are collected by the central office of statistics in Denmark, which are collecting all of society’s statistical information and are used for research, education ect. The data are anonymized and are situated on private servers owned by the *Danmarks Statistik*. These data are, by Danish law, not public available and data are thus not possible to share.

## Abbreviations

AF: Atrial fibrillation
CIs: Confidence intervals
ICD: International Classification of disease
IR: Incidence rates
IRRs: Incidence rate ratios
PY: Person-years
SD: Standard deviation
