# Supplementary material for: Incidence of atrial fibrillation in different major cancer subtypes: a Nationwide population-based 12 year follow up study
Source: BMC Cancer. 2019 Nov 14;19:1105. doi: 10.1186/s12885-019-6314-9 (PMC6854796; doi:10.1186/s12885-019-6314-9)
Supplement: Supplementary file 1 — Additional file 1: Table S1. Incidence rate ratios of atrial fibrillation in overall cancer and in different cancer types divided into different time periods. The model is adjusted for time, age, sex, comorbidities and earlier surgeries. [file 12885_2019_6314_MOESM1_ESM.docx]

Additional file 1: **Table S1.** Incidence rate ratios of atrial fibrillation in overall cancer and in different cancer types divided into different time periods. The model is adjusted for time, age, sex, comorbidities and earlier surgeries.

|  | 0-90 days | p-value | 90-180 days | p-value | 180-365 days | p-value | 1-2 years | p-value | 2-5 years | p-value | >5 years | p-value |
| --- | --- | --- | --- | --- | --- | --- | --- | --- | --- | --- | --- | --- |
| All cancer | 4.07 (3.94-4.21) |  | 2.14 (2.03-2.25) |  | 1.58 (1.52-1.66) |  | 1.30 (1.25-1.35) |  | 1.12 (1.09-1.16) |  | 1.09 (1.05-1.12) |  |
| N | 3831 |  | 1486 |  | 1929 |  | 2545 |  | 4583 |  | 3773 |  |
| Lung cancer | 8.66 (8.12-9.23) | <0.0001 | 3.65 (3.25-4.10) | <0.0001 | 3.28 (2.97-3.63) | <0.0001 | 2.24 (1.99-2.51) | <0.0001 | 1.66 (1.48-1.86) | <0.0001 | 1.02 (0.86-1.21) | 0.8022 |
| N | 943 |  | 290 |  | 380 |  | 285 |  | 293 |  | 133 |  |
| Breast cancer | 2.68 (2.35-3.07) | <0.0001 | 1.17 (0.95-1.44) | 0.1353 | 1.08 (0.93-1.26) | 0.2949 | 1.23 (1.11-1.36) | 0.0001 | 1.10 (1.02-1.18) | 0.0089 | 1.14 (1.06-1.22) | 0.0003 |
| N | 214 |  | 91 |  | 168 |  | 355 |  | 786 |  | 786 |  |
| Colon cancer | 3.10 (2.84-3.39) | <0.0001 | 1.57 (1.32-1.87) | <0.0001 | 1.42 (1.23-1.63) | <0.0001 | 1.13 (1.00-1.28) | 0.0472 | 1.09 (1.00-1.19) | 0.0615 | 1.07 (0.98-1.18) | 0.1345 |
| N | 581 |  | 123 |  | 195 |  | 254 |  | 506 |  | 456 |  |
| Prostate cancer | 1.90 (1.69-2.15) | <0.0001 | 1.21 (1.04-1.41) | 0.0148 | 1.17 (1.04-1.30) | 0.0068 | 1.13 (1.04-1.23) | 0.0042 | 1.06 (1.00-1.13) | 0.0539 | 1.06 (0.98-1.14) | 0.1268 |
| N | 260 |  | 164 |  | 310 |  | 539 |  | 1088 |  | 679 |  |
| Cancer in the uterus, cervix or in the ovaries | 4.80 (4.07-5.66) | <0.0001 | 1.83 (1.38-2.43) | <0.0001 | 1.15 (0.89-1.49) | 0.2280 | 1.26 (1.04-1.53) | 0.0165 | 1.16 (1.02-1.32) | 0.291 | 1.26 (1.11-1.42) | 0.0003 |
| N | 142 |  | 48 |  | 57 |  | 106 |  | 218 |  | 255 |  |
| Urinary tract cancer | 2.97 (2.58-3.42) | <0.0001 | 1.85 (1.53-2.25) | <0.0001 | 1.48 (1.26-1.73) | <0.0001 | 1.28 (1.12-1.47) | 0.0004 | 1.03 (0.92-1.51) | 0.5788 | 0.96 (0.85-1.09) | 0.5651 |
| N | 192 |  | 103 |  | 152 |  | 205 |  | 319 |  | 247 |  |
| Hematological cancer | 5.95 (5.36-6.60) | <0.0001 | 2.91 (2.49-3.40) | <0.0001 | 1.72 (1.48-2.00) | <0.0001 | 1.48 (1.31-1.68) | <0.0001 | 1.26 (1.15-1.38) | <0.0001 | 1.28 (1.16-1.42) | <0.0001 |
| N | 359 |  | 158 |  | 176 |  | 254 |  | 468 |  | 393 |  |
| Upper gastrointestinal cancer | 5.03 (4.38-5.76) | <0.0001 | 4.58 (3.81-5.52) | <0.0001 | 2.91 (2.36-3.61) | <0.0001 | 1.70 (1.32-2.19) | <0.0001 | 1.37 (1.08-1.74) | 0.0108 | 1.08 (0.80-1.46) | 0.6191 |
| N | 211 |  | 112 |  | 85 |  | 61 |  | 66 |  | 43 |  |
| Rectal cancer | 3.17 (2.80-3.59) | <0.0001 | 2.35 (1.97-2.80) | <0.0001 | 1.52 (1.27-1.82) | <0.0001 | 1.18 (1.00-1.39) | 0.045 | 1.09 (0.97-1.23) | 0.1671 | 1.02 (0.90-1.16) | 0.7632 |
| N | 263 |  | 125 |  | 120 |  | 142 |  | 269 |  | 225 |  |
| Cancer in the liver, pancreas or gallbladder | 3.65 (3.11-4.28) | <0.0001 | 2.12 (1.66-2.95) | <0.0001 | 1.77 (1.31-2.39) | 0.0002 | 1.33 (0.94-1.87) | 0.1034 | 1.00 (0.69-1.44) | 0.9897 | 1.11 (0.71-1.72) | 0.6513 |
| N | 155 |  | 47 |  | 43 |  | 33 |  | 29 |  | 20 |  |
| Skin cancer | 3.50 (2.83-4.34) | <0.0001 | 2.03 (1.52-2.70) | <0.0001 | 1.29 (0.99-1.67) | 0.0602 | 1.14 (0.92-1.40) | 0.2260 | 1.10 (0.95-1.27) | 0.1993 | 1.11 (0.96-1.28) | 0.1470 |
| N | 83 |  | 46 |  | 56 |  | 88 |  | 185 |  | 197 |  |
| Cancer in the central nervous system | 8.08 (6.37-10.25) | <0.0001 | 4.30 (2.86-6.48) | <0.0001 | 1.98 (1.20-3.29) | 0.0080 | 1.23 (0.68-2.22) | 0.4950 | 0.44 (0.21-0.93) | 0.0307 | 0.78 (0.44-1.37) | 0.3795 |
| N | 68 |  | 23 |  | 15 |  | 7 |  | 12 |  | 12 |  |
| Endocrin cancer | 3.68 (2.09-6.49) | <0.0001 | 1.32 (0.50-3.51) | 0.5805 | 1.50 (0.75-3.00) | 0.2502 | 1.19 (0.66-2.16) | 0.5569 | 0.93 (0.60-1.44) | 0.7476 | 0.88 (0.56-1.37) | 0.5621 |
| N | 12 |  | 4 |  | 8 |  | 11 |  | 20 |  | 19 |  |
| Other cancer | 4.02 (3.62-4.47) | <0.0001 | 2.52 (2.15-2.95) | <0.0001 | 1.61 (1.38-1.88) | <0.0001 | 1.28 (1.11-1.47) | 0.0005 | 1.10 (0.991.21) | 0.0780 | 0.96 (0.86-1.08) | 0.4897 |
| N | 348 |  | 152 |  | 164 |  | 201 |  | 372 |  | 308 |  |
