# Supplementary material for: Incidence of atrial fibrillation in different major cancer subtypes: a Nationwide population-based 12 year follow up study
Source: BMC Cancer. 2019 Nov 14;19:1105. doi: 10.1186/s12885-019-6314-9 (PMC6854796; doi:10.1186/s12885-019-6314-9)
Supplement: Supplementary file 2 — Additional file 2: Table S2. Incidence rate ratios of atrial fibrillation in overall cancer divided into different time periods. Atrial fibrillation here only as the primary diagnosis. The model is adjusted for time, age, sex, comorbidities and earlier surgeries. [file 12885_2019_6314_MOESM2_ESM.docx]

Additional file 2: **Table S2**. Incidence rate ratios of atrial fibrillation in overall cancer divided into different time periods. Atrial fibrillation here only as the primary diagnosis. The model is adjusted for time, age, sex, comorbidities and earlier surgeries.

|  | 0-90 days | p-value | 90-180 days | p-value | 180-365 days | p-value | 1-2 years | p-value | 2-5 years | p-value | >5 years | p-value |
| --- | --- | --- | --- | --- | --- | --- | --- | --- | --- | --- | --- | --- |
| Cancer  n | 3.02 (2.85-3.20) | <0.0001 | 1.72 (1.58-1.87) | <0.0001 | 1.41 (1.32-1.51) | <0.0001 | 1.15 (1.08-1.22) | <0.0001 | 1.05 (1.01-1.10) | 0.0196 | 1.00 (0.96-1.05) | 0.9127 |
|  | 1260 |  | 573 |  | 840 |  | 1106 |  | 2048 |  | 1657 |  |
