# Supplementary material for: Incidence of atrial fibrillation in different major cancer subtypes: a Nationwide population-based 12 year follow up study
Source: BMC Cancer. 2019 Nov 14;19:1105. doi: 10.1186/s12885-019-6314-9 (PMC6854796; doi:10.1186/s12885-019-6314-9)
Supplement: Supplementary file 3 — Additional file 3: Table S3. Incidence rate ratios of atrial fibrillation in overall cancer and in the different cancer types. The model is adjusted for time, age, sex, comorbidities and earlier surgeries. [file 12885_2019_6314_MOESM3_ESM.docx]

Additional file 3: **Table S3**. Incidence rate ratios of atrial fibrillation in overall cancer and in the different cancer types. The model is adjusted for time, age, sex, comorbidities and earlier surgeries.

| Type of cancer | Incidence rate ratios | p-value |
| --- | --- | --- |
| All cancer  n | 1.46 (1.44 – 1.48) | <0.0001 |
|  | 18,147 |  |
| Lung cancer  n | 3.16 (3.04-3.30) | <0.0001 |
|  | 2324 |  |
| Breast cancer  n | 1.20 (1.16-1.25) | <0.0001 |
|  | 2357 |  |
| Colonic cancer  n | 1.35 (1.29-1.41) | <0.0001 |
|  | 2115 |  |
| Prostate cancer  n | 1.14 (1.10-1.18) | <0.0001 |
|  | 3040 |  |
| Cancer in the uterus, cervix or the ovaries  n | 1.43 (1.33-1.53) | <0.0001 |
|  | 826 |  |
| Urinary tract cancer  n | 1.29 (1.22-1.36) | <0.0001 |
|  | 1218 |  |
| Hematological cancer  n | 1.71 (1.63-1.79) | <0.0001 |
|  | 1808 |  |
| Upper gastrointestinal cancer  n | 2.54 (2.34-2.75) | <0.0001 |
|  | 578 |  |
| Rectal cancer  n | 1.38 (1.30-1.46) | <0.0001 |
|  | 1144 |  |
| Skin cancer  n | 1.28 (1.19-1.38) | <0.0001 |
|  | 655 |  |
| Central nervous system cancer  n | 2.22 (1.88-2.63) | <0.0001 |
|  | 136 |  |
| Cancer in the liver, pancreas or gallbladder  n | 1.99 (1.78-2.22) | <0.0001 |
|  | 327 |  |
| Endocrine cancer  n | 1.16 (0.93-1.46) | 0.1962 |
|  | 74 |  |
| Other cancers  N | 1.45 (1.38-1.52) | <0.0001 |
|  | 1545 |  |
