# Supplementary material for: Incidence of atrial fibrillation in different major cancer subtypes: a Nationwide population-based 12 year follow up study
Source: BMC Cancer. 2019 Nov 14;19:1105. doi: 10.1186/s12885-019-6314-9 (PMC6854796; doi:10.1186/s12885-019-6314-9)
Supplement: Supplementary file 4 — Additional file 4. ICD and ATC codes for identification of cancer diagnosis, surgeries, comorbidities and pharmacotherapy. [file 12885_2019_6314_MOESM4_ESM.docx]

Codes for identification of cancer diagnosis, surgeries, comorbidities and pharmacotherapy

**Code**

**Cancer types (ICD codes)**

Upper GI cancer DC15, DC16, DC17

Colonic cancer DC18

Rectum cancer DC20, DC21

Liver, galdbladder, pancreas DC22, DC23, DC24, DC25

Lung cancer DC34

Skin cancer DC43

Breast cancer DC50

Cancer in cervix, uterine and the ovaries DC53, DC54, DC55, DC56

Prostate cancer DC61

Urinary tract cancer DC64, DC65, DC66, DC67, DC68

CNS cancer DC70, DC71, DC72

Endocrine cancer DC73, DC74

Heamatological cancer DC81, DC82, DC83, DC84, DC85, DC86, DC87, DC88, DC89, DC90, DC91, DC92, DC93, DC94, DC95, DC96

Other cancer DC00-DC14, DC19, DC26-DC33, DC37-DC41, DC45-DC49, DC51, DC52, DC57, DC58, DC60, DC62, DC63, DC69, DC75

Not included DC44

**Surgeries**

Cardiacsurgeries KF* (excluding KFNG, KFJA, KFFA, KFNF, KFLA, KFFA, KFEE, KFW, KFX, KFP)

Gastricsurgeries KJC-KJN* (excluding KJE, KJH, KJK)

Orthosurgeries KN* (excluding KNB, KNC, KND, KNH)

**Comorbidities**

Stroke **ICD10:** DI63, DI64, DG458, DG459
**ICD8:** 438

Ischeamic heart disease **ICD10:** DI21, DI22, DI702-DI709, DI700
**ICD8:** 410

All emboulus **ICD10:** DI26, DI63, DI64, DI74, DG458, DG459
**ICD8:** 433-438, 444, 450

Bleeding **ICD10:** DI60-DI62, DN02, DR31, DR04, DD50, DD62, DK250, DK252, DK252, DK254, DK260, DK262, DK264, DK270, DK272, DK280, DK282, DK920-DK922, DS064-DS066, DJ942
**ICD8:** 430-432

Vascular disease **ICD10:** DI21, DI22, DI700, DI702-DI709
**ICD8:** 410, 440

Hypertension **ATC:** C02A-C02C, C02DA, C02DB, C02DD, C02DG, C02L, C03A, C03B, C03D, C03E, C03X, C07A-C07D, C07F, C08, C08G, C09AA, C09BA, C09BB, C09CA, C09DA, C09DB, C09XA02, C09XA52

Heart failure **ICD10:** I110, I42, I50, J819

**ICD8:** 425, 4270, 4271

**ATC:** C03C

Renal disease **ICD10:** DN02-DN08, DN11, DN12, DN14, DN18, DN19, DN26, DN158, DN159, DQ612, DQ613, DQ615, DQ619, DE102, DE112, DE132, DE142, DI120, DN160, DN162-DN164, DN168, DM300, DM313, DM319
**ICD8:** 403, 404, 580-584, 590,223, 25002, 75310, 75311, 75319

Diabetes mellitus **ATC:** A10

Liver **ICD10:** DB15-DB19, DB22, DC22, DK70-DK77, DZ944, DD684C, DQ618A
**ICD8:** 571-573, 155, 070

Alcohol **ICD10:** DE52, DE244, FD1, DG312, DG621, DG721, DK70, DK292, DK860, DI426, DL278A, DO354, DT51, DZ714, DZ721
**ICD8**: 291, 303

Hyperthyroid disease **ICD10:** DE04, DE05

Chronic obstrutive pulmonary disease **ICD10:** DJ44

**Pharmacotherapy (ATC codes)**

Betablocker C07

ACE* C09

Loop diuretics CO3C

Thiazid diuretics C03A

Spironolactone C03D

Calcium channel blockers C08

Warfarin B01AA03

Aspirin B01AC06

Clopidogrel B01AA04

Digoxin C01AA05

Lipid lowering drugs C10A

Glucose-lowering medication A10

Inhalation R03

*Nordic classification of surgical procedure codes
